# miR-19 regulates the expression of interferon-induced genes and MHC class I genes in human cancer cells

**DOI:** 10.7150/ijms.44377

**Published:** 2020-04-06

**Authors:** Jing Li, Tao-Yan Lin, Lin Chen, Yu Liu, Mei-Juan Dian, Wei-Chao Hao, Xiao-Lin Lin, Xiao-Yan Li, Yong-Long Li, Mei Lian, Heng-Wei Chen, Jun-Shuang Jia, Xiao-Ling Zhang, Sheng-Jun Xiao, Dong Xiao, Yan Sun

**Affiliations:** 1 Guangdong Provincial Key Laboratory of Cancer Immunotherapy Research and Guangzhou Key Laboratory of Tumor Immunology Research, Cancer Research Institute, Southern Medical University, Guangzhou 510515, China; 2 Zhongshan School of Medicine, Sun Yat-sen University, Guangzhou 510080, China; 3 Institute of Comparative Medicine & Laboratory Animal Center, Southern Medical University, Guangzhou 510515, China; 4 Department of Pharmacy, Nanfang Hospital, Southern Medical University, Guangzhou 510515, China; 5 Radiotherapy Center, the First People's Hospital of Chenzhou, Chenzhou 423000, China; 6 Department of Physiology, Faculty of Basic Medical Sciences, Guilin Medical University, Guilin 541004, China; 7 Department of Pathology, the Second Affiliated Hospital, Guilin Medical University, Guilin 541199, China

**Keywords:** miR-19a, miR-19b-1, lung cancer, nasopharyngeal carcinoma, MHC class I gene, interferon-inducible gene, interleukin-related gene

## Abstract

MicroRNA-19 (miR-19) is identified as the key oncogenic component of the miR-17-92 cluster. When we explored the functions of the dysregulated miR-19 in lung cancer, microarray-based data unexpectedly demonstrated that some immune and inflammatory response genes (i.e., IL32, IFI6 and IFIT1) were generally down-regulated by miR-19 overexpression in A549 cells, which prompted us to fully investigate whether the miR-19 family (i.e., miR-19a and miR-19b-1) was implicated in regulating the expression of immune and inflammatory response genes in cancer cells. In the present study, we observed that miR-19a or miR-19b-1 overexpression by miRNA mimics in the A549, HCC827 and CNE2 cells significantly downregulated the expression of interferon (IFN)-regulated genes (i.e., IRF7, IFI6, IFIT1, IFITM1, IFI27 and IFI44L). Furthermore, the ectopic miR-19a or miR-19b-1 expression in the A549, HCC827, CNE2 and HONE1 cells led to a general downward trend in the expression profile of major histocompatibility complex (MHC) class I genes (such as HLA-B, HLA-E, HLA-F or HLA-G); conversely, miR-19a or miR-19b-1 inhibition by the miRNA inhibitor upregulated the aforementioned MHC Class I gene expression, suggesting that miR-19a or miR-19b-1 negatively modulates MHC Class I gene expression. The miR-19a or miR-19b-1 mimics reduced the expression of interleukin (IL)-related genes (i.e., IL1B, IL11RA and IL6) in the A549, HCC827, CNE2 or HONE1 cells. The ectopic expression of miR-19a or miR-19b-1 downregulated IL32 expression in the A549 and HCC827 cells and upregulated IL32 expression in CNE2 and HONE1 cells. In addition, enforced miR-19a or miR-19b-1 expression suppressed IL-6 production by lung cancer and nasopharyngeal carcinoma (NPC) cells. Taken together, these findings demonstrate, for the first time, that miR-19 can modulate the expression of IFN-induced genes and MHC class I genes in human cancer cells, suggesting a novel role of miR-19 in linking inflammation and cancer, which remains to be fully characterized.

## Introduction

MicroRNAs (miRNAs) are implicated in cancer initiation, progression, metastasis and angiogenesis 1. The miR-17-92 gene cluster is frequently amplified in B-cell lymphomas and in a wide range of solid tumors, including lung and stomach cancers^1^, ^2^. The miR-19 family (including miR-19a and miR-19b-1) is considered a key component of the oncogenic miR-17-92 cluster^2^.

Our previous study demonstrated that miR-19 triggered epithelial-mesenchymal transition (EMT), accompanied by the reduced proliferation of lung cancer cells^3^. When we explored the roles of dysregulated miR-19 in lung cancer, microarray-based gene expression data unexpectedly demonstrated a significant number of inflammatory and immune response genes up- or down-regulated by miR-19, including interferon(IFN)-stimulated genes such as GBP1, IFRD1, IFI35, IFI6 and PSMB9, and complement components, suggesting a strong relationship between miR-19 and inflammation in cancer. On the other hand, there are several lines of evidence that miR-19 family can act as regulators of inflammatory response in some diseases, including rheumatoid arthritis (RA)^4^, ^5^, asthma^6^, atherosclerosis^7^, ^8^, nasal polyposis^9^ and sepsis^10^. miR-17-92 was down-regulated in activated fibroblast-like synoviocytes (FLS) isolated from RA patients, and miR-19a and miR-19b-1 regulate Toll- like receptor 2 (TLR2) expression thereby reducing the inflammatory response induced by bacterial lipoprotein (BLP) in FLS and which is characterized by the secretion of IL-6 and Matrix metalloproteinases (MMP-3) 4, 11. miR-19a upregulation in asthma is an indicator and a cause of increased T_H_2 cytokine production in the airways^6^. Moreover, the expression of interferon- regulated genes and MHC class I molecules are modulated by microRNAs, such as miR-9^12^, miR-125a^13^, miR-520b^14^, let-7f-5p 15, let-7i-5p^15^, miR-146b-5p^15^ and miR-185-5p 15. Our findings and published reports strongly indicate a novel role of miR-19 in linking inflammation and cancer, which remains to be fully characterized.

These aforementioned findings prompted us to fully explore whether the miR-19 family was involved in regulating the expression of IFN-related genes, major histocompatibility complex (MHC) class I genes and interleukin (IL)-related genes in cancer cells.

## Materials and Methods

### Cell line and cell culture

Human NPC cell lines (i.e., CNE2 and HONE1) were cultured in Roswell Park Memorial Institute 1640 medium (RPMI1640) (Corning) and lung cancer cell lines (i.e., A549 and HCC827) were cultured in Dulbecco's modified Eagle's medium (DMEM) (Corning), respectively, supplemented with 10% fetal bovine serum (FBS) (Biological Industries) in a humidified incubator with 5% CO_2_ at 37°C. All cells were approved by the Institutional Review Board of Southern Medical University.

### miRNA transient transfection

Human miR-19 mimics (including miR-19a mimics and miR-19b-1 mimics), a nonspecific miRNA control (i.e., mimics control), human miR-19 inhibitors (including miR-19a inhibitor and miR-19b-1 inhibitor) and a nonspecific miRNA inhibitor control were purchased from Shanghai GenePharma Co., Ltd. (Shanghai, China). miRNA inhibitor which is a chemically modified single strand of RNA can sequester miRNAs avoiding their targeting to coding genes of endogenous target miRNA. miRNAs were transiently transfected into cells at a working concentration of 100 nmol/L using Lipofectamine 2000 reagent (Invitrogen) in accordance with the manufacturer's procedure. Cancer cells (i.e., A549, HCC827, CNE2 or HONE1 cells) were transfected with miR-19 mimics (100 nM) or miR-19 inhibitor (100 nM) for 48h, respectively, followed by evaluating the expression of the indicated IFN-inducible genes, MHC class I genes or IL-related genes via qRT-PCR.

### Cytokine production assay

Lung cancer cells (A549 and HCC827) and NPC cells (CNE2 and HONE1) were transfected with 100 nM miR-19 mimics or its mimics control. 24 hours and 48 hours after culture, cell culture supernatants were collected and assayed for supernatant concentrations of IL6 by ELISA kit (MultiSciences, China) according to the manufacturer's instructions.

### RNA isolation, reverse transcription and qRT-PCR

For miRNA and mRNA analyses, total RNA from cancer cells was extracted with Trizol Reagent (TaKaRa). Total RNA was reversely transcribed with the PrimeScript RT reagent Kit (TaKaRa). The expression levels of mature miRNA were determined by SYBR Green quantitative PCR amplifications performed on the Stratagene Mx3005P Real-Time PCR system (Agilent Technologies, Inc., USA). U6 was used for internal reference genes for miRNA^16^. Expression of mRNA analysis was performed using SYBR Green Master Mix (TaKaRa), and GAPDH was used for normalization. The primers used for the amplification of the indicated genes were listed in Table 1-4.

### mRNA microarray analysis

As described in our paper^3^, miR-19- and vector-expressing A549 cells were analyzed by Affymetrix arrays. Expression microarray analysis was carried out with commercially available Affymetrix Human Gene U133 Plus 2.0 array according to the Affymetrix standard protocol, which carries 47,000 transcripts representing 38,500 well-characterized human genes. Data analysis was performed using the Significance Analysis of Microarray software (SAM 3.0, Stanford University, USA; http://www-stat. stanford.edu). Heatmap is plotted using pheatmap R package.

### Statistical analysis

Data were presented as mean±SD unless otherwise indicated of at least 3 independent experiments. Statistical analysis was performed using a SPSS 16.0 software (SPSS, North Chicago, IL) software package. Statistical significance was assessed by one-way analysis of variance (ANOVA) (*P<0.5; ^#^*P*<0.01).

## Results

### miR-19 overexpression and downregulated endogenous miR-19 expression in cancer cells

**Figure 1:**
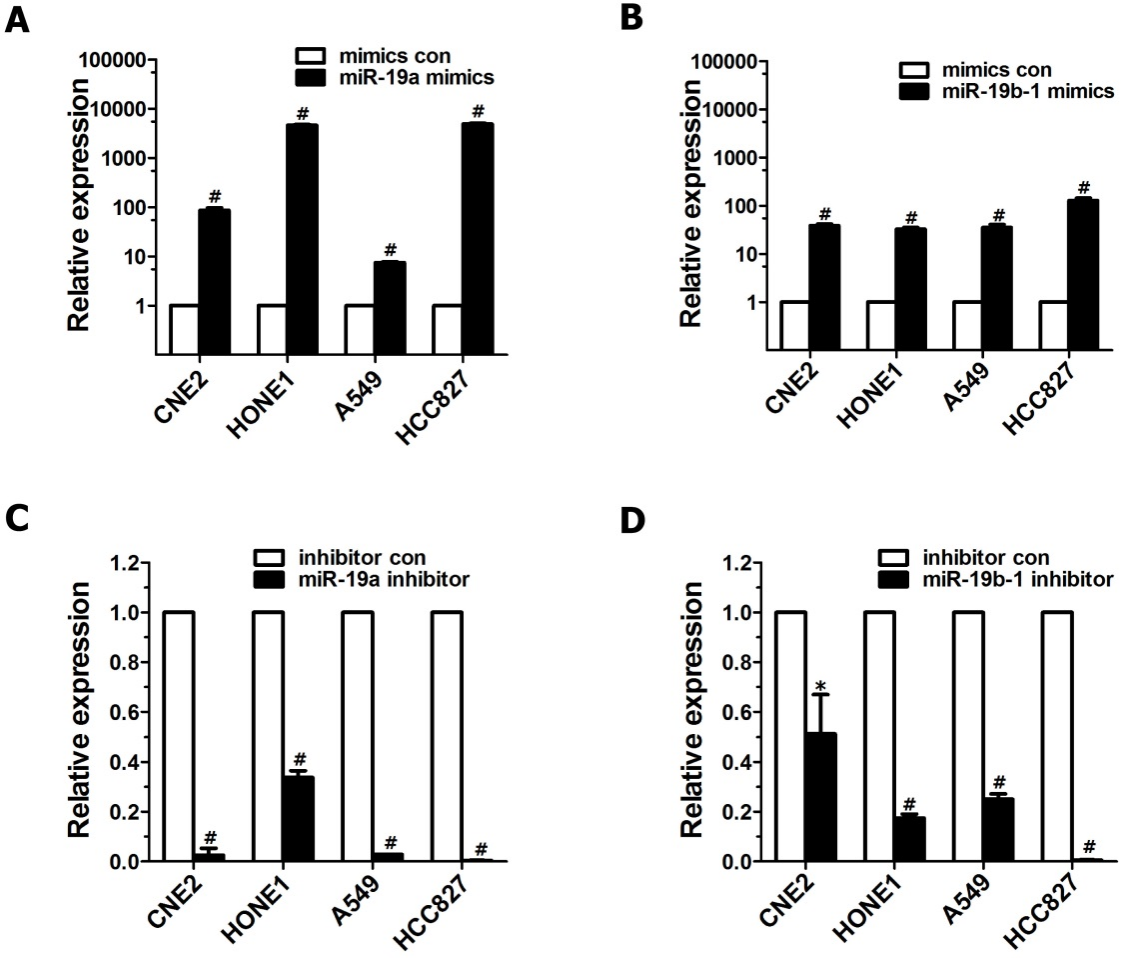
** miR-19 overexpression and down-regulated endogenous miR-19 expression in human cancer cells.** qRT-PCR-based assay was used to analyze the expression levels of miR-19 after human lung adenocarcinoma cell lines (A549 and HCC827) and NPC cell lines(CNE2 and HONE1) transfected with miR-19 mimics (100 nM) (**A-B**) or miR-19 inhibitor (100 nM) (**C-D**) for 48 hours (h). *P<0.5, ^#^*P*<0.01 by one-way ANOVA.

To explore the roles of miR-19 dysregulation in the pathogenesis of lung cancer and NPC, two methods were employed to overexpress miR-19 in cancer cells. First, A549 cells expressing both miR-19a and 19b-1 transgenes were simultaneously generated as described in our previous publication^3^. Next, miR-19a or miR-19b-1 mimics were transiently transfected into the indicated cancer cells. As shown in Figure 1, the levels of miR-19a (Figure 1A) or miR-19b-1 (Figure 1B) in indicated cells transfected with miR-19a or miR-19b-1 mimics were much higher than those in cancer cells transfected with control mimics. Moreover, the levels of miR-19a (Figure 1C) or miR-19b-1 (Figure 1D) in the indicated cancer cells transfected with the miR-19a or miR-19b-1 inhibitor were much lower than those in cells transfected with the control inhibitor.

### Global analysis of gene expression changes in miR-19-expressing A549 cells

**Figure 2:**
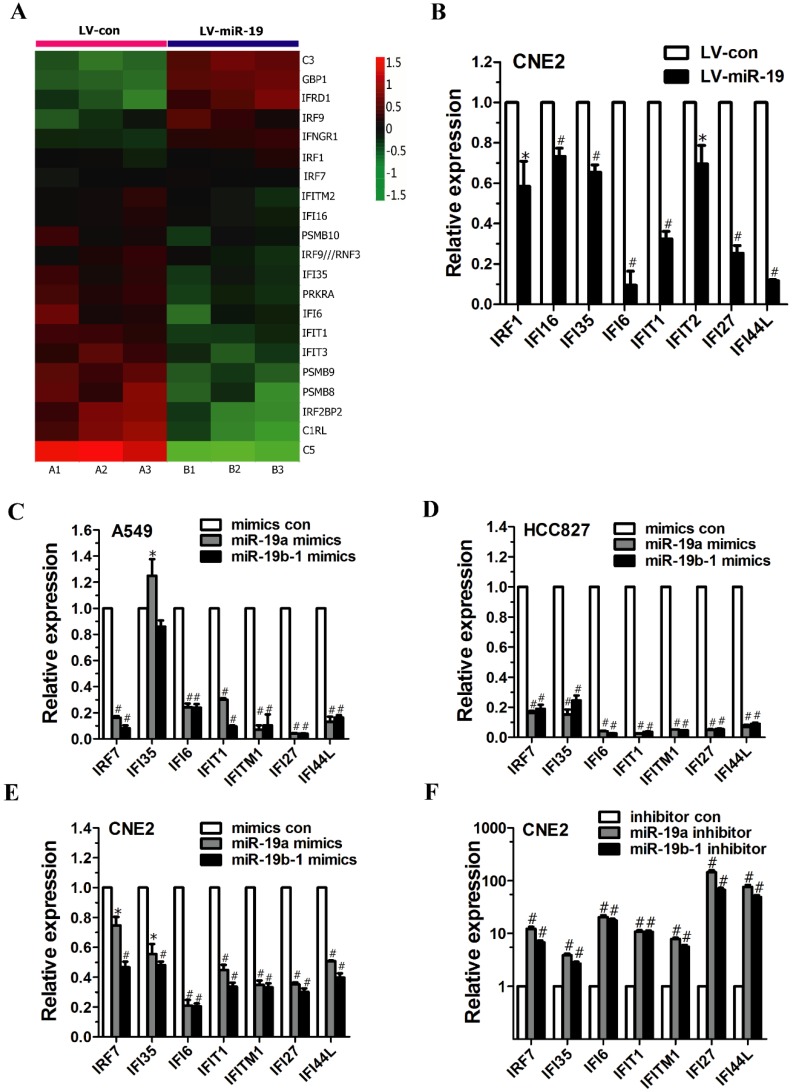
** miR-19 regulated the expression of IFN-related genes in cancer cells.** (**A**) Class comparison and hierarchical clustering of differentially expressed IFN-regulated genes between miR-19- and vector-expressing A549 cells. In the cluster heatmap of IFN-inducible gene, A1, A2 and A3 represented the total RNA isolated from different generations of vector-expressing A549 cells, and B1, B2 and B3 represented the total RNA isolated from different generations of miR-19-expressing A549 cells. Genes with increased and reduced expressions are shown in red and green, respectively. (**B**) qRT-PCR analyzed IFN-regulated gene expression in CNE2 cells expressing miR-19a and 19b-1 transgenes simultaneously. To generate stable cell line, the recombinant lentiviruses (i.e., LV-con and LV-miR-19) were used to infect CNE2 cells to generate vector- and miR-19-expressing CNE2 cells. (**C-D**) miR-19 modulated IFN-related gene expression in A549 (C) and HCC827 (D) cells. (**E-F**) miR-19 regulated IFN-related gene expression in CNE2 cells. *P<0.5, ^#^*P*<0.01 by one-way ANOVA.

The global gene expression changes induced by miR-19 overexpression were determined by comparing the gene expression profiles between miR-19- and vector-expressing A549 cells based on microarray data. We found that a total of 352 and 501 genes were significantly downregulated and upregulated, respectively, by miR-19 overexpression 3. Unexpectedly, among the 853 significantly changed genes, we found that some of the differentially expressed genes were involved in regulating immune and/or inflammatory responses, including IFN-inducible genes (Figure 2A, and Table 5) and IL-related genes (Figure 4A). Table 6 lists the GO terms representing biological processes related to immune and inflammatory responses. In summary, the microarray data suggest that miR-19 plays a significant role in regulating the expression of genes involved in immune and inflammatory responses.

### miR-19 altered IFN-regulated gene expression in cancer cells

As mentioned above, microarray analysis revealed the altered expression of IFN-regulated genes (e.g., GBP1, IRF9, IFI35, PRKRA, IFI6, IFIT1, IFIT3, PSMB9 and IRF2BP2) (Figure 2A). Moreover, qRT-PCR analysis demonstrated the significantly decreased expression of IFN-regulated genes (e.g., IRF1, IFI16, IFI35, IFI6, IFIT1, IFIT2, IFI27 and IFI44L) in CNE2 cells expressing both miR-19a and 19b-1 transgenes (Figure 2B).

To characterize the effects of the individual components of the miR-19 family on the IFN-regulated target genes, cancer cells were transfected with miR-19a (Figure 1A) and miR-19b-1 mimics (Figure 1B). As shown in Figure 2C and D, miR-19a and miR-19b-1 mimics significantly downregulated the expression of IFN-regulated genes (i.e., IRF7, IFI6, IFIT1, IFITM1, IFI27 and IFI44L) in both A549 and HCC827 cells. Next, we further explored whether miR-19 overexpression by miR-19a or miR-19b-1 mimics alters the expression of IFN-regulated genes in other cancer cells. qRT-PCR data revealed a general downward trend in the expression of IFN-regulated genes (i.e., IRF7, IFI35, IFI6, IFIT1, IFITM1, IFI27 and IFI44L) in CNE2 cells transfected with miR-19a or miR-19b-1 mimics (Figure 2E); conversely, endogenous miR-19 inhibition by the miR-19a or miR-19b-1 inhibitor in CNE2 cells correspondingly resulted in the upregulated expression of the aforementioned IFN-regulated genes (Figure 2F). Overall, miR-19a or miR-19b-1 overexpression leads to a general downward trend in the expression profile of the aforementioned genes involved in IFN induction in cancer cells.

### Suppression of MHC class I gene expression by miR-19 in cancer cells

**Figure 3:**
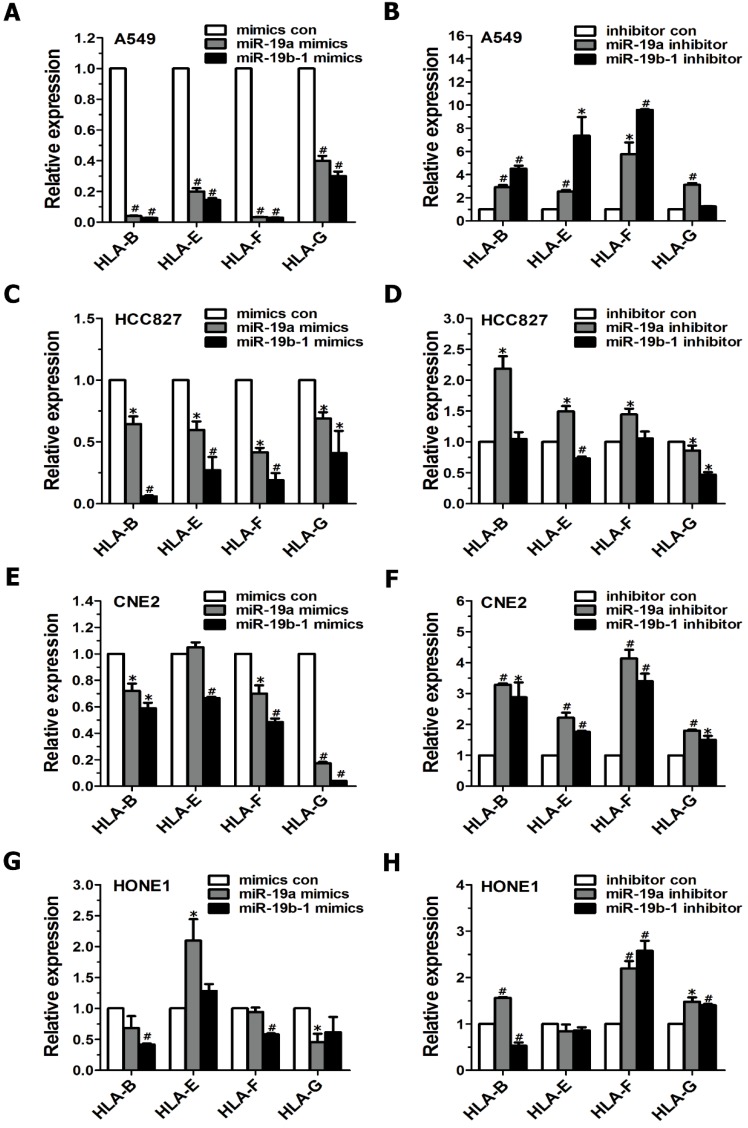
**miR-19 modulated the expression of MHC class I genes in cancer cells.** Cancer cells were transfected with miR-19 mimics (100 nM)(A,C,E,G) or miR-19 inhibitor (100 nM)(B,D,F,H) for 48h, respectively, followed by evaluating MHC class I gene expression via qRT-PCR.

Although MHC class I molecules are constitutively expressed in essentially all nucleated cells, the cumulative evidence shows that tumor cells display downregulated MHC class I antigens, which enables them to evade immune surveillance^17^. Therefore, we further examined the influence of the miR-19 family on MHC class I gene expression in cancer cells. When cancer cells (i.e., A549, HCC827, CNE2 and HONE1 cells) were transiently transfected with miR-19a or miR-19b-1 mimics, qRT-PCR data revealed a general downward trend in the expression profile of the MHC Class I genes (such as HLA-B, HLA-F, HLA-G) (Figure 3A, C, E, G). Conversely, these tumor cells, when transfected with the miR-19a or miR-19b-1 inhibitor, showed a general upward trend in the indicated MHC Class I genes (Figure 3B, D, F, H). Taken together, the most significant change after the enforced miR-19a or miR-19b-1 expression in cancer cells was the generally decreased expression of the abovementioned MHC Class I genes.

### miR-19 overexpression enhanced or reduced IL-related gene expression in cancer cells

**Figure 4:**
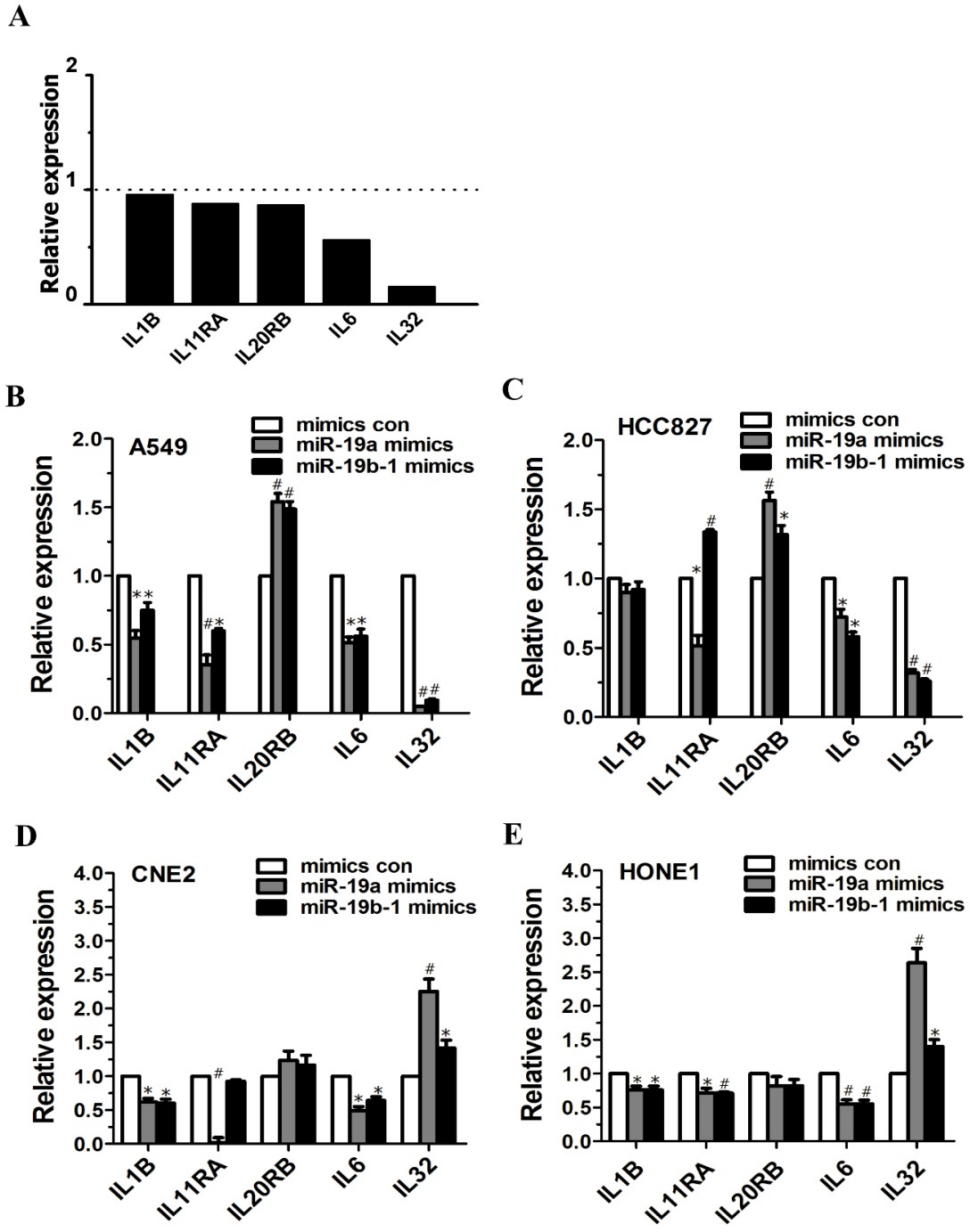
**miR-19 overexpression enhanced or reduced the IL-related genes expression in cancer cells.** (**A**) Graph illustrating the fold change in gene expression of representative differentially IL-related genes between LV-miR-19-infected A549 cells to LV-con-infected A549 cells. The horizontal dashed line marks a fold change of 1 (no change). (**B-E**) miR-19a or miR-19b-1 overexpression altered the IL-related gene expression in cancer cells. *P<0.5, ^#^*P*<0.01 by one-way ANOVA.

Microarray data demonstrated altered expression of IL6 (fold change: 0.5616) and IL32 (fold change: 0.1536) (Figure 4A). miR-19a or miR-19b-1 overexpression led to the downregulation of IL1B expression in A549, CNE2 and HONE1 cells (Figure 4B, D, E), and miR-19a overexpression reduced IL11RA expression in A549, HCC827, CNE2 and HONE1 cells (Figure 4B, C, D, E). The ectopic expression of miR-19a or miR-19b-1 upregulated IL20RB expression in A549 and HCC827 cells (Figure 4B, C) but not in CNE2 and HONE1 cells (Figure 4D, E). Moreover, miR-19a or miR-19b-1 overexpression reduced IL6 expression in the lung cancer and NPC cells examined (Figure 4B, C, D, E). miR-19a or miR-19b-1 mimics significantly downregulated IL32 expression in A549 and HCC827 cells (Figure 4B, C) and remarkably upregulated IL32 expression in CNE2 and HONE1 cells (Figure 4D, E). Together, ectopic miR-19a or miR-19b-1 expression alters IL-related gene expression.

### miR-19 overexpression resulted in decreased IL6 production by cancer cells

**Figure 5:**
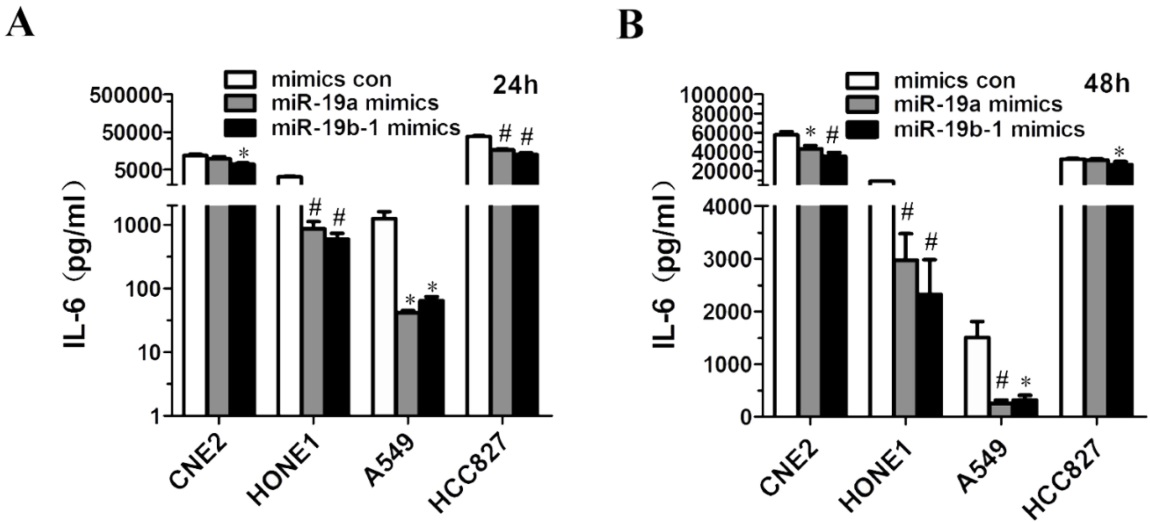
**miR-19a or miR-19b-1 overexpression resulted in the decreased IL6 production by cancer cells.** The indicated cells were transfected with 100 nM miR-19 mimics or its mimics control, and incubated for 24h (**A**) and 48h (**B**). Supernatants were collected and assayed for IL6 levels. *P<0.5, ^#^*P*<0.01 by one-way ANOVA.

Given that miR-19a or miR-19b-1 mimics suppressed IL6 expression in lung cancer and NPC cells (Figure 4B, C, D, E), we further investigated the possibility that ectopic miR-19a or miR-19b-1 expression could affect IL6 production by these cancer cells. Strikingly, a marked decrease in the production of IL6 was observed in A549, HCC827, CNE2 and HONE1 cells 24 h (Figure 5A) and 48 h (Figure 5B) after mimic transfection. In summary, these findings demonstrate that miR-19 can modulate the IL6 production in cancer cells.

## Discussion

Over the last few years, there has been increasing evidence that miR-19a or miR-19b-1 positively regulates cancer cell proliferation and tumorigenesis 18-20, stemness^21^, and EMT, invasion and metastasis^18^-^20^ by targeting different targets, suggesting that miR-19 plays oncogenic roles in cancer progression. Our microarray data unexpectedly revealed immune and inflammatory response genes down- or upregulated by miR-19 overexpression in lung cancer, which encouraged us to investigate the functions of onc-miR-19 in linking inflammation and cancer.

The miR-17-92 cluster regulates a broad spectrum of biological processes of T cell immunity, and is found to facilitate T cell proliferation, enhance antitumor activities and promote T cell-dependent antibody responses^22^. Moreover, some studies revealed the roles of miR-17-92 in the development of autoimmune diseases^22^, ^23^. miR-17-92 which is part of the CD28 costimulatory network regulated IL-10 production by Foxp3^+^ Tregs and control of experimental autoimmune encephalomyelitis (EAE)^23^. T cell-specific miR-17-92 deficiency reduced T_H_17 differentiation, which consequently ameliorated EAE symptoms^22^. Further studies demonstrated that miR-19b-1 is one of miRNAs in miR-17-92 cluster responsible for promoting T_H_17-mediated inflammation by targetedly repressing PTEN expression, thereby augmenting the PI3K-AKT-mTOR axis essential for proper T_H_17 differentiation^22^.

Increasing evidence that miR-19 family (i.e., miR-19a and miR-19b-1) has been highly involved in immunity^4^ and inflammatory diseases, including RA^4^, ^5^, ^11^, asthma^6^, ^24^, ^25^, atherosclerosis^7^, ^8^, diabetic retinopathy^26^, nasal polyposis^9^, sepsis 10, encephalitis^27^ and Crohn's disease^28^-^31^. miR-19b-1 positively regulated NF-κB signaling, which is one of the critical promoters of inflammation, while the positive regulation of NF-κB signaling by miR-19b-1 involves the coordinated suppression of negative regulators of NF-κB signaling, including A20/Tnfaip3, Rnf11, Fbxl11/Kdm2a and Zbtb16^4^. miR-17-92 was down-regulated in activated fibroblast-like synoviocytes (FLS) isolated from RA patients (RAFLS), miR-19b-1 enhanced basal IL6 and IL8 secretion by RAFLS to increase basal inflammation, and miR-19a and miR-19b-1 regulate IL6 and MMP-3 release by controlling TLR2 expression^4^, ^5^, ^11^. Elevated expression of miR-19a in human airway-infiltrating T cells of patients with asthma, and miR-19a promoted T_H_2 cytokine production in the airways and amplified inflammatory signaling by direct targeting of the inositol phosphatase PTEN, the signaling inhibitor SOCS1 and the deubiquitinase A20 24, while miR-19b-1 reduced airway remodeling, airway inflammation and degree of oxidative stress by inhibiting Stat3 signaling through TSLP downregulation in a mouse asthma model^25^. Moreover, the endothelial hypoxia-inducible factor-1α promoted atherosclerosis and monocyte recruitment by upregulating miR-19a^7^, while TNF-α or IFN-γ or IL-4 suppressed IL-10 in B cells (from patients with atherosclerosis) via upregulating miR-19a expression 8. miR-19a negatively interfered with IL-10 expression in peripheral dendritic cells (DCs) of patients with nasal polyposis^19^. miR-19a directly regulated TNF-α expression in ulcerative colitis^28^, and miR-19b-1 suppressed the inflammatory response by inhibiting SOCS3 to modulate chemokine production in intestinal epithelial cells (IECs) and thereby prevented the pathogenesis of Crohn's disease (CD)^29^. Japanese encephalitis virus-mediated inflammation via miR-19b-1, which plays important roles in immunity and inflammatory diseases.

Chronic inflammation is believed to have a crucial role in cancer development, and inflammation not only works as a tumor-promoting agent but also influences other steps of tumorigenesis by inducing DNA damage, angiogenesis, invasion and metastasis 32, 33. There is increasing evidence that miRNAs drive tumor progression by regulating inflammation 34, 35. The inhibition of miR-9, which was overexpressed in Hodgkin lymphoma, decreased the production of cytokines (i.e., TNF-α, CCL-5, IL-6 and IL-5) from L428 and L540 cells of Hodgkin lymphoma, followed by an impaired ability to attract normal inflammatory cells and then impairing tumor outgrowth *in vivo*^36^. Additionally, our previous study revealed that the most significant change following tumor suppressor miR-9 overexpression in NPC cells was the decreased expression of IL-related genes, including IL1B, IL11, IL1F8, IL1A, IL6 and IL7R 12. miR-19a promoted colitis and colitis-associated colon cancer by downregulating TNFAIP3 in a targeted manner and constitutively activating NF-κB signaling^27^. miRNA-19a/b-1 exhibits oncogenic activity through negatively regulating SOCS3 via the JAK-STAT pathway together with the increased activation of SOCS3, IL6 and STAT3^37^. In this study, we revealed that ectopic expression of miR-19a or miR-19b-1 modulated the expression of IL-related genes (including IL1B, IL11RA, IL20RB, IL6 and IL32) and suppressed IL6 production in lung cancer and NPC cells. We found that miR-19a or miR-19b-1 overexpression resulted in decreased IL1B expression in lung cancer and NPC cells, while microglia-derived IL1B triggered p53-mediated cell cycle arrest and apoptosis in neural precursor cells^38^, indicating that reduced IL1B expression induced by onco-miR-19 might contribute to cancer progression. In summary, these findings strongly indicate a novel role of miR-19 in linking inflammation and cancer, which remains to be characterized.

IL32 is a novel cytokine involved in inflammation and cancer development^39^. Various published data demonstrated that IL32 promotes or decreases tumor development^39^. For example, IL32 contributes to gastric cancer progression by increasing the metastatic potential resulting from AKT, β-catenin and HIF-1α activation^40^. IL-32 inhibits tumor growth via inhibition of NF-κB and STAT3 signals in colon and prostate cancer^39^. In this study, we found that miR-19a or miR-19b-1 overexpression remarkably downregulated IL32 expression in lung cancer cells and led to an increase in IL32 expression in NPC cells. These findings from this study and other published reports strongly suggest that modulating IL32 expression by miR-19 might contribute to the pathogenesis of lung cancer and NPC, which remains to be examined.

IL6, a multifunctional cytokine, is involved in the host immune defense mechanism as well as the modulation of growth and differentiation in various malignancies^41^. The deregulated overexpression of IL6 is associated with tumor progression through inhibition of cancer cell apoptosis, stimulation of angiogenesis and drug resistance^41^. Clinical studies revealed that increased serum IL6 concentrations in patients are associated with advanced tumor stages of various cancers (e.g., lung and colorectal cancers) and short survival in patients^41^. Thus, blocking IL6 signaling is a potential therapeutic strategy for cancers (i.e., anti-IL6 therapy) characterized by pathological IL6 overproduction. miR-19a and miR-19b-1 repressed the release of the cytokines IL6 and MMP-3 in BLP-activated RAFLS by controlling TLR2 expression, suggesting that miR-19a/b-1 can act as negative regulators of inflammation in humans^11^. Additionally, our present study revealed that miR-19a or miR-19b-1 overexpression reduced IL6 expression and production in cancer cells, which contradicts the pathological IL6 overproduction observed in various cancers described above. In summary, the aforementioned findings encourage us to further explore how the decreased IL6 expression and production resulting from the miR-19 overexpression in lung cancer and NPC cells contributes to cancer progression.

Increasing evidence supports that the IFN-inducible genes that are expressed in tumor cells regulate cancer cell proliferation and tumorigenesis^42^ and metastasis^43^. IFITM1 is a negative regulator of cell proliferation and tumorigenesis in hepatocellular carcinoma (HCC), while the anti-proliferative action of interferon-γ is mediated by IFITM1^42^. The silencing of IRF7 in breast cancer cells promoted bone metastasis through tumor immune escape, while the IRF7-driven suppression of metastasis was reliant on IFN signaling to host immune cells 43. In this study, we revealed for the first time that the ectopic expression of miR-19a or miR-19b-1 in cancer cells significantly downregulated the expression of PSMB8, PSMB9 and IFN-regulated genes (i.e., IRF7, IFI6, IFIT1, IFITM1, IFI27 and IFI44L), which have never been reported in other physiological and pathological processes. Additionally, the expression of IFN-induced protein family members with tetratricopeptide repeats (IFIT), including IFIT1, IFIT2, IFIT3 and IFIT5, was decreased in HCC tissues^44^. Higher IFIT3 expression in HCC tissues predicts a better response to IFN-a therapy in HCC patients, and IFIT3 promotes IFN-a effector responses and therapeutic effects by strengthening IFN-a effector signaling in HCC^44^. In summary, these findings from this study and other published reports strongly suggest that modulating the expression of IFN-inducible genes in cancer cells by miR-19 might contribute to the pathogenesis of lung cancer and NPC, which remains to be thoroughly investigated.

MHC class I molecules, including the classical MHC class I molecules [i.e., HLA(human leukocyte antigen)-A, HLA-B and HLA-C] and non-classical MHC class I molecules (i.e., HLA-E, HLA-F, HLA-G, HLA-H and HLA-J), are constitutively expressed in essentially all nucleated cells^17^. Many cancer cells display downregulated MHC class I antigen (MHC-I), which enables them to evade immune surveillance, while the downregulation of HLA class I expression contributes to a poor prognosis in cancer patients 17. The MHC class I promoter is known to be activated by several transcription factors, including CIITA^45^-^48^ and USF1^49^, ^50^. CXCR4-mediated cell surface MHC-I downregulation in cancer progression facilitated tumor evasion of immune surveillance^51^. Kinases, including MAP2K1 (MEK1), EGFR and RET, were validated as negative regulators of human HLA expression in multiple cancer types, and activated MAPK signaling in mouse tumors *in vivo* suppressed components of MHC-I, while the pharmacologic inhibition of MAPK signaling led to improved peptide/MHC target recognition and killing by T cells and TCR-mimic antibodies^52^. EGFR tyrosine kinase inhibitors augmented the expression of MHC-I and MHC-II molecules in primary and malignant human keratinocytes^53^. The inhibition of the MAPK pathway induced the upregulation of HLA-A expression and enhanced the sensitivity of targeted tumors to Ag-specific CTL lysis in esophageal and gastric cancer^54^. Additionally, miR-9, which functions as a tumor suppressor in NPC^55^, positively regulated the expression of MHC class I genes (such as HLA-B and HLA-F) in NPC^12^. The previous studies revealed that the non-classical HLA-F and HLA-G act as the important mediators of immune escape^56^, ^57^. This study demonstrated that miR-19a or miR-19b-1 overexpression in cancer cells led to a general downward trend in the expression profile of MHC Class I molecules (such as HLA-B, HLA-E, HLA-F, HLA-G or HLA-J), which has never been reported in other physiological and pathological processes. As described in the discussion section, miR-19a and miR-19b-1 function as oncomiR in various cancer progressions. Taken together, these findings shed new light on the role of the miR-19 family in tumor evasion of immune surveillance by inducing the downregulation of cell surface HLA class I expression, which remains to be fully characterized.

## Conclusion

We have demonstrated that miR-19 can regulate IL-related gene expression in lung cancer cells and NPC cells, and we have found for the first time that miR-19 overexpression and/or inhibition alter the expression of IFN-induced genes and MHC class I genes in cancer cells. However, the pathological consequences of the altered expression of immune- or inflammatory-related genes by miR-19 in cancer cells remain to be examined. Future studies are required to clarify the potential contribution of miR-19 to the proliferation, EMT, invasion, metastasis or tumor angiogenesis of cancer cells by playing critical roles in modulating the aforementioned genes involved in immune and inflammation responses. Collectively, we suspect that miR-19 might function as an oncomiR to promote tumor progression by regulating the expression of the above-mentioned genes involved in immunity and inflammation.

## Figures and Tables

**Table 1:** Primers for qRT-PCR analysis of human miR-19

Primer name	Primer sequence
U6 snRNA-RT	AACGCTTCACGAATTTGCGT
U6 snRNA-forward primer	CTCGCTTCGGCAGCACA
U6 snRNA-reverse primer	AACGCTTCACGAATTTGCGT
miR-19-RT-primer	GTCGTATCCAGTGCAGGGTCCGAGGTATTCGCACTGGATACGACTCAGT
miR-19a-forward primer	TGTGCAAATCTATGCAAA
miR-19a-reverse primer	GTGCAGGGTCCGAGGTATTC
miR-19b-1-forward primer	TGTGCAAATCCATGCAAA
miR-19b-1-reverse primer	GTGCAGGGTCCGAGGTATTC

**Table 2:** Primers for qRT-PCR analysis of interferon-regulated genes

Gene	Forward primer (5'-3')	Reverse primer (5'-3')
IFI6	GGTCTGCGATCCTGAATGGG	TCACTATCGAGATACTTGTGGGT
IFI16	AGACTGAAGACTGAACCTGAAGA	GAACCCATTGCGGCAAACATA
IFI27	TGCTCTCACCTCATCAGCAGT	CACAACTCCTCCAATCACAACT
IFI35	GTGGACGTTCGGGAGCTAC	ACTGGCCGATTTGGCACAG
IFI44L	AGCCGTCAGGGATGTACTATAAC	AGGGAATCATTTGGCTCTGTAGA
IFIT1	TTGATGACGATGAAATGCCTGA	CAGTCACCAGACTCCTCAC
IFIT2	AAGCACCTCAAAGGGCAAAAC	TCGGCCCATGTGATAGTAGAC
IFITM1	CCAAGGTCCACCGTGATTAAC	ACCAGTTCAAGAAGAGGGTGTT
IRF1	ATGCCCATCACTCGGATGC	CCCTGCTTTGTATCGGCCTG
IRF7	CCCACGCTATACCATCTACCT	GATGTCGTCATAGAGGCTGTTG
GAPDH	ACAACTTTGGTATCGTGGAAGG	GCCATCACGCCACAGTTTC

**Table 3:** Primers for qRT-PCR analysis of MHC class I genes

Gene	Forward Primer (5'-3')	Reverse Primer (5'-3')
HLA-B	CAGTTCGTGAGGTTCGACAG	CAGCCGTACATGCTCTGGA
HLA-E	TTCCGAGTGAATCTGCGGAC	GTCGTAGGCGAACTGTTCATAC
HLA-F	TGGCCCTGACCGATACTTG	GCAGGAATTGCGTGTCGTC
HLA-G	GAGGAGACACGGAACACCAAG	GTCGCAGCCAATCATCCACT

**Table 4:** Primers for qRT-PCR analysis of interleukin-related genes

Gene	Forward Primer (5'-3')	Reverse Primer (5'-3')
IL1B	CAGCTACGAATCTCCGACCAC	GGCAGGGAACCAGCATCTTC
IL6	ACTCACCTCTTCAGAACGAATTG	CCATCTTTGGAAGGTTCAGGTTG
IL11RA	CAGCCAGATCAGCGGTTTAC	AGATGCTCTGCAAGCTCACAT
IL20RB	GGCCACTGTGCCATACAAC	TCTTTGGTGATCTCCATCCCA
IL32	AGCTGGAGGACGACTTCAAA	AGAGCAGCAGAAACTCTGGA

**Table 5:** Differentially expressed interferon-regulated genes

Gene symbol	Annotation	Fold change
C3	complement component 3	2.0557
GBP1	guanylate binding protein 1, interferon-inducible, 67kDa	2.0482
IFRD1	interferon-related developmental regulator 1	1.9062
IRF9	interferon regulatory factor 9	1.5356
IFNGR1	interferon gamma receptor 1	1.4824
IRF1	interferon regulatory factor 1	1.1651
IRF7	interferon regulatory factor 7	1.0475
IFI16	interferon, gamma-inducible protein 16	0.8606
IFITM2	interferon induced transmembrane protein 2 (1-8D)	0.8439
PSMB10	proteasome (prosome, macropain) subunit, beta type, 10	0.7610
IRF9 /// RNF31	interferon regulatory factor 9 /// ring finger protein 31	0.7605
IFI35	interferon-induced protein 35	0.6960
PRKRA	protein kinase, interferon-inducible double stranded RNA dependent activator	0.6525
IFI6	interferon, alpha-inducible protein 6	0.6417
IFIT1	interferon-induced protein with tetratricopeptide repeats 1	0.6346
IFIT3	interferon-induced protein with tetratricopeptide repeats 3	0.6016
PSMB9	proteasome (prosome, macropain) subunit, beta type, 9 (large multifunctional peptidase 2)	0.5405
PSMB8	proteasome (prosome, macropain) subunit, beta type, 8 (large multifunctional peptidase 7)	0.5074
IRF2BP2	interferon regulatory factor 2 binding protein 2	0.4720
C1RL	complement component 1, r subcomponent-like	0.4445
C5	complement component 5	0.2388

**Table 6:** Gene ontology analysis of up- and down-regulated genes (related with immune and inflammation) from miR-19-expressing A549 cells to vector-expressing A549 cells

GO ID	Biological process	Count	*p*-value	*q*-value
GO:0006954	inflammatory response	20	1.83E-25	5.78E-25
GO:0006958	complement activation, classical pathway	4	1.60E-07	1.01E-07
GO:0045087	innate immune response	4	1.00E-04	2.77E-05
GO:0006955	immune response	8	1.21E-04	3.28E-05
GO:0006957	complement activation, alternative pathway	2	2.74E-04	6.69E-05
GO:0030101	natural killer cell activation	2	5.45E-04	1.19E-04
GO:0002842	positive regulation of T cell mediated immune response to tumor cell	1	0.003256	4.26E-04
GO:0002378	immunoglobulin biosynthesis	1	0.003256	4.26E-04
GO:0002862	negative regulation of inflammatory response to antigenic stimulus	1	0.00488	5.70E-04
GO:0002863	positive regulation of inflammatory response to antigenic stimulus	1	0.006502	6.77E-04
GO:0050729	positive regulation of inflammatory response	1	0.033668	0.002274
GO:0045089	positive regulation of innate immune response	1	0.036815	0.002446
GO:0002768	immune response-regulating cell surface receptor signaling pathway	1	0.044637	0.002855

## Abbreviations

qRT-PCR: quantitative real-time polymerase chain reaction
IFN: interferon
MHC: major histocompatibility complex
IL: interleukin
NPC: nasopharyngeal carcinoma
